# Unsupervised clustering algorithms improve the reproducibility of dynamic contrast-enhanced magnetic resonance imaging pulmonary perfusion quantification in muco-obstructive lung diseases

**DOI:** 10.3389/fmed.2022.1022981

**Published:** 2022-10-24

**Authors:** Marilisa Konietzke, Simon M. F. Triphan, Monika Eichinger, Sebastian Bossert, Hartmut Heller, Sabine Wege, Ralf Eberhardt, Michael U. Puderbach, Hans-Ulrich Kauczor, Gudula Heußel, Claus P. Heußel, Frank Risse, Mark O. Wielpütz

**Affiliations:** ^1^Boehringer Ingelheim Pharma GmbH and Co. KG, Biberach an der Riß, Germany; ^2^Department of Diagnostic and Interventional Radiology, Subdivision of Pulmonary Imaging, University Hospital of Heidelberg, Heidelberg, Germany; ^3^Translational Lung Research Center Heidelberg (TLRC), German Center for Lung Research (DZL), Heidelberg, Germany; ^4^Department of Diagnostic and Interventional Radiology With Nuclear Medicine, Thoraxklinik at the University Hospital of Heidelberg, Heidelberg, Germany; ^5^Department of Pulmonology and Respiratory Medicine, Thoraxklinik at the University Hospital of Heidelberg, Heidelberg, Germany; ^6^Department of Diagnostic and Interventional Radiology, Hufeland Hospital, Bad Langensalza, Germany

**Keywords:** muco-obstructive lung disease, functional imaging, contrast agent lung perfusion, cystic fibrosis (CF), chronic obstructive pulmonary disease (COPD)

## Abstract

**Background:**

Dynamic contrast-enhanced magnetic resonance imaging (DCE-MRI) allows the assessment of pulmonary perfusion, which may play a key role in the development of muco-obstructive lung disease. One problem with quantifying pulmonary perfusion is the high variability of metrics. Quantifying the extent of abnormalities using unsupervised clustering algorithms in residue function maps leads to intrinsic normalization and could reduce variability.

**Purpose:**

We investigated the reproducibility of perfusion defects in percent (QDP) in clinically stable patients with cystic fibrosis (CF) and chronic obstructive pulmonary disease (COPD).

**Methods:**

15 CF (29.3 ± 9.3y, FEV1%predicted = 66.6 ± 15.8%) and 20 COPD (66.5 ± 8.9y, FEV1%predicted = 42.0 ± 13.3%) patients underwent DCE-MRI twice 1 month apart. QDP, pulmonary blood flow (PBF), and pulmonary blood volume (PBV) were computed from residue function maps using an in-house quantification pipeline. A previously validated MRI perfusion score was visually assessed by an expert reader.

**Results:**

Overall, mean QDP, PBF, and PBV did not change within 1 month, except for QDP in COPD (*p* < 0.05). We observed smaller limits of agreement (± 1.96 *SD*) related to the median for QDP (CF: ± 38%, COPD: ± 37%) compared to PBF (CF: ± 89%, COPD: ± 55%) and PBV (CF: ± 55%, COPD: ± 51%). QDP correlated moderately with the MRI perfusion score in CF (*r* = 0.46, *p* < 0.05) and COPD (*r* = 0.66, *p* < 0.001). PBF and PBV correlated poorly with the MRI perfusion score in CF (*r* =−0.29, *p* = 0.132 and *r* =−0.35, *p* = 0.067, respectively) and moderately in COPD (*r* =−0.57 and *r* =−0.57, *p* < 0.001, respectively).

**Conclusion:**

In patients with muco-obstructive lung diseases, QDP was more robust and showed a higher correlation with the MRI perfusion score compared to the traditionally used perfusion metrics PBF and PBV.

## Introduction

Patients with muco-obstructive lung diseases such as cystic fibrosis (CF) and chronic obstructive pulmonary disease (COPD) show regional impairment of pulmonary perfusion due to hypoxic pulmonary vasoconstriction (HPV) in response to alveolar hypoxia. In CF and COPD, alveolar hypoxia is caused by the obstruction of conducting airways, small airway obliteration, and due to destruction of lung parenchyma and the alveolar-capillary bed. Regional pulmonary perfusion abnormalities become apparent on dynamic contrast-enhanced magnetic resonance imaging (DCE-MRI) as absent or delayed contrast enhancement. A semi-quantitative, morpho-functional MRI scoring system, including an MRI perfusion score, has been established and validated in previous studies (1–6). Furthermore, the reproducibility of the MRI perfusion score in clinically stable patients with CF and COPD has been demonstrated (7).

The automated assessment of perfusion abnormalities by computer algorithms is possible either by directly quantifying pulmonary blood flow (PBF) and pulmonary blood volume (PBV) or, more recently, by quantifying the extent of pulmonary perfusion abnormalities in percent relative to the lung volume (perfusion defects in percent, QDP) (8–12). These automated assessments can help to address inter-reader variability issues with human readers (1, 7), facilitate detailed perfusion analyses and are time efficient.

PBF and PBV have been used to assess pulmonary perfusion directly for the whole lung (11, 13). However, this includes also a regional perfusion increase in response to blood flow redistribution due to HPV, thereby compensating a regional perfusion decrease in poorly ventilated lung regions. This may result in an underestimation of pathology for the whole lung, which limits the usefulness of this approach for monitoring disease progression or determining severity (14). In contrast, QDP quantifies only the extent of perfusion defects but not redistribution effects at a local (voxel) level, which leads to an intrinsic normalization. In fact, perfusion defects can be better delineated by the QDP approach when an increase in perfusion caused by the HPV in well-ventilated areas occurs. Initial evidence suggests that QDP might be superior to PBF and PBV, as a cross-sectional study demonstrated better correlations with lung function assessed by spirometry and with emphysema and functional small airway disease assessed by quantitative CT (12).

Determining a method’s precision including its reproducibility is a prerequisite before a biomarker can be used in clinical drug development studies. Until now, the reproducibility of PBF and PBV has been studied in patients with COPD or healthy subjects, but not in patients with CF. These studies showed limited reproducibility of PBF and PBV, which is probably caused by low contrast-to-noise ratios (CNR), low temporal resolution, non-linearities of the contrast agent concentration to signal relationship, differences in the inspiratory level, pronounced image artifacts in DCE-MRI of the lungs, and in one study due to residual circulating contrast agent (15–19). The recently introduced QDP method has the potential to overcome challenges of quantitative lung DCE-MRI, particularly with respect to variability of the assessment. However, the reproducibility and robustness of QDP has not yet been investigated.

In this study, two consecutive standardized DCE-MRI examinations were conducted 1 month apart in patients with CF and COPD in stable clinical condition. The objectives of this work were to assess (a) the midterm reproducibility of QDP, (b) the association between QDP and the visual MRI perfusion score, and (c) the association between QDP and spirometry. All QDP evaluations were performed in comparison to PBF and PBV.

## Materials and methods

### Study population

This prospective observational study in patients with CF and COPD was approved by the institutional ethics committee (S-126/2015), and informed written consent for examination and data evaluation was obtained from all subjects (7). Patients with a hypersensitivity to gadolinium-based contrast agents were not enrolled. Both MRI examinations were performed in stable clinical condition, which was defined as constant maintenance therapy and freedom from pulmonary exacerbation as defined elsewhere (20, 21). Patients experiencing a pulmonary exacerbation or a major change in symptoms or therapy after the first MRI needed to return to baseline medication and symptoms at latest 7 days prior to second MRI. Further details are provided with the online data supplement (Supplementary Table 1).

### MRI acquisition

MRI was performed at baseline (MRI1) and 1 month (30.0 ± 2.5 days) later (MRI2) using the same 1.5T scanner (Magnetom Aera, Siemens Healthineers, Erlangen, Germany) and protocol (Supplementary Table 2) as reported previously (4, 7, 22). The perfusion DCE-MRI was acquired with a time-resolved 3D gradient echo sequence with parallel imaging and view sharing (time-resolved angiography with stochastic trajectories [TWIST]). Morphological MR image for the lung segmentation was acquired using a T1-weighted gradient echo sequence (GRE) with volume interpolated breath-hold acquisition (VIBE). Further details are provided with the online data supplement.

### Chest MRI score

All MRI examinations were assessed using a well-validated chest MRI scoring system, including the MRI perfusion score (1, 3, 5, 6, 23, 24), by a reader with more than 12 years of experience in chest MR. The results of the chest MRI score in this cohort have been reported previously, and the MRI perfusion score did not change significantly between MRI1 and MRI2 for CF and COPD (7).

### Automated quantification of pulmonary perfusion abnormalities

All quantitative MRI perfusion parameters were computed with an in-house developed MRI quantification pipeline written in MATLAB (R2019a, The MathWorks, Inc., Natick, MA, United States), as described previously (12). In brief, time-resolved subtraction images from DCE-MRI were generated by subtracting the mean of the first two pre-contrast images. Arterial input functions (AIF) were automatically determined in the pulmonary artery (25). The lungs were automatically segmented from morphological MR images using a region growing approach, which were registered to the DCE-MRI (12, 26). Each lung segmentation was reviewed by an analyst. The performance of the underlying lung segmentation principles was investigated in a previous study by comparing automatically segmented lungs with manually segmented lungs performed by two chest radiologists and has shown excellent agreement (26). The quantification of perfusion abnormalities was performed on time-resolved residue function maps (R(t) maps), which were calculated by deconvolving each voxel of the subtraction image with the AIF using truncated singular value decomposition (27). For the QDP quantification, the R(t) maps at the time of maximum contrast enhancement (Rmax maps) were median filtered (square 5 × 5 neighborhood) to reduce noise and remove isolated voxels while preserving edges. Otsu’s method, an unsupervised histogram-based image clustering algorithm, was employed to the median filtered Rmax maps for the calculation of QDP. Two thresholds from the signal histogram of the entire Rmax map were determined by Otsu’s method to classify the voxels per scan into three different clusters (poorly perfused, well-perfused and vessels). QDP reflects the voxels in the poorly perfused cluster, which includes the voxels with the lowest contrast enhancement. Therefore, all voxels of the individual Rmax map with signal intensities below the lowest threshold determined by Otsu’s method was classified as QDP. QDP was quantified in percent representing the extent of perfusion abnormalities relative to the segmented lung volume per patient. The gravity effect on local perfusion due to the patient’s supine position in the MRI scanner was accounted for in the QDP calculation by adapting the threshold for each slice (8, 10). The quantification of the conventional pulmonary perfusion metrics PBF and PBV was performed voxel-wise on the R(t) maps. PBF is defined as the maximum height of the residue function in each voxel and PBV as time-integral of the residue function in each voxel (13, 27). PBF and PBV were calculated for the whole lung by taking the median from all voxels within the lungs.

### Spirometry

Spirometry (MasterScreen Body, E. Jaeger, Hochberg, Germany) was performed according to the American Thoracic Society and European Respiratory Society recommendations at the same days as the MRI examinations (28). In this work, the forced expiratory volume in 1s percent predicted (FEV1%predicted) was used.

As published previously, in this study mean FEV1%predicted did not change significantly from MRI1 to MRI2 in CF and in COPD (7).

### Statistical analysis

Data were analyzed using R project for statistical computing (R 3.3.2 Foundation for Statistical Computing, Vienna, Austria). Data are presented as mean ± standard deviation (*SD*). Bland-Altman analysis including limits of agreement (LoA = ± 1.96*SD), linear regression, Spearman correlation, minimal important difference (MID) based on standard error of measurement from repeated measures analysis of variance (rANOVA), Wilcoxon signed-rank test, Kruskal-Wallis test, Pearson and Filon’s z, and scatterplots were used (29–33). A *p*-value < 0.05 was considered statistically significant. Further details are provided with the online data supplement.

## Results

### Patient population and technical feasibility

**FIGURE 1:**
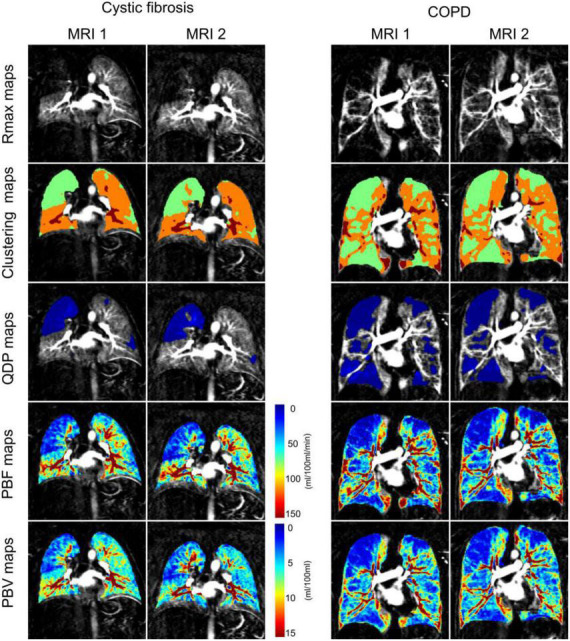
Coronal residue function maps at the time point of maximum contrast-enhancement in the whole lung (Rmax maps), corresponding clustering maps for perfusion defects in percent maps (QDP maps) computation (3 clusters from 2 thresholds determined with Otsu’s method), QDP maps (defects highlighted in blue) and pulmonary blood flow maps (PBF maps) and pulmonary blood volume maps (PBV maps) from a representative cystic fibrosis and chronic obstructive pulmonary disease (COPD) patient at baseline (MRI1) and 1 month follow-up (MRI2). QDP was 21.57% at MRI1 and 20.61% at MRI2 in the cystic fibrosis patient, and 42.96% at MRI1 and 43.56% at MRI2 in the COPD patient (highlighted in blue). Perfusion scores were 8 at MRI1 and 7 at MRI2 for the cystic fibrosis patient, and 12 at MRI1 and 12 at MRI2 for the COPD patient.

In total, 35 patients, 15 with CF (29.3 ± 9.3y, FEV1%predicted = 66.6 ± 15.8%) and 20 with COPD (66.5 ± 8.9y, FEV1%predicted = 42.0 ± 13.3%) completed both MRI examinations in stable clinical condition (Figure 1 and Table 1).

**TABLE 1:** Patient demographics.

	Cystic fibrosis	COPD	Both groups
***N* =**	15	20	35
**Age (y)**	29.3 ± 9.3	66.5 ± 8.9	50.6 ± 20.7
**Sex**	2 f/13 m	5 f/15 m	7 f/28 m
**History of smoking, n**	2	20	22
**Pack years**	0.7 ± 0.2	52.3 ± 22.2	47.6 ± 26.8
**Time since diagnosis (y)**	20.9 ± 11.7	8.1 ± 5.5	13.6 ± 10.7
**BMI (kg/m^2^)**	21.8 ± 2.8	25.1 ± 3.7	23.7 ± 3.7
***P. aeruginosa* status**	5 Chronic 4 Intermittent	–	–
***S. aureus* status**	9	–	–
***Aspergillus* ssp. status**	4 Chronic 4 Intermittent	–	–

Data presented as mean ± standard deviation. The differences between both patient cohorts, i.e., cystic fibrosis versus COPD, and the baseline data of the MRI scores and spirometry have been previously reported in a research letter elsewhere (7).

BMI, body mass index; COPD, chronic obstructive pulmonary disease. Of note, bacterial colonization was not routinely assessed in COPD.

In four MRI exams (5.7%, three at MRI1 and one at MRI2) from three different patients (one CF patient and two COPD), the MRI perfusion score could not be visually scored due to contrast bolus mistiming or insufficient contrast enhancement.

Quantitative perfusion analysis was not performed on 11 MRI exams (15.7%, six at MRI1 and five at MRI2) from eight different patients (two CF patients and six COPD patients) due to contrast bolus mistiming, insufficient contrast enhancement or substantial breathing artifacts. 32 DCE-MRI series (59%) were refined by manually removing acquisitions at time points with respiratory artifacts before or after the contrast agent bolus passage through the lung.

### Quantitative perfusion parameters are reproducible in clinically stable cystic fibrosis and chronic obstructive pulmonary disease patients

**FIGURE 2:**
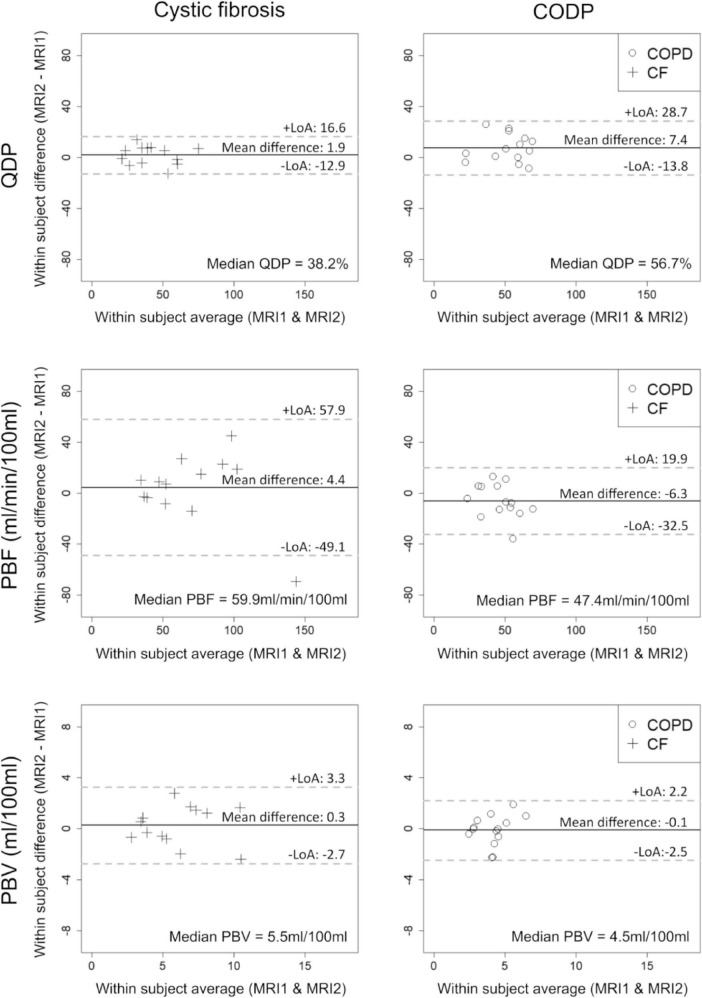
Bland-Altman plots showing the midterm reproducibility of pulmonary perfusion defects in percent (QDP), pulmonary blood flow (PBF) and pulmonary blood volume (PBV) in adults with clinically stable cystic fibrosis (CF, crosses) and chronic obstructive pulmonary disease (COPD, circles). 13 CF patients and 14 COPD patients were evaluated. Mean differences, limits of agreement (LoA) and median values are given for each panel. Solid lines indicate the mean difference between MRI1 and MRI2, dashed lines the LoA (± 1.96*SD).

Out of the three quantitative MRI perfusion parameters evaluated in this study, only QDP increased significantly in COPD from MRI1 to MRI2 with a mean difference of 7.4 (LoA = ± 21.2, *p* < 0.05). In CF, QDP tended to increase with a mean difference of 1.9 (LoA = ± 14.7, *p* = 0.273). In contrast, PBF and PBV had a tendency to increase (*p* = 0.216 and *p* = 0.542, respectively) in CF and to decrease from MRI1 to MRI2 in COPD (Figure 2 and Table 2).

**TABLE 2:** Reproducibility of quantitative pulmonary perfusion parameters in clinically stable cystic fibrosis and COPD patients.

	Cystic fibrosis			COPD		
	MRI1	MRI2	MRI1 vs. MRI2 p	MRI1	MRI2	MRI1 vs. MRI2 p
***N* =**		13			14	
**Mean ± *SD***						
QDP	39.1 ± 18.9	42.8 ± 15.8	0.273	48.7 ± 15.9	54.3 ± 17.4	<0.05
PBF (ml/100 ml/min)	75.8 ± 47.9	70.9 ± 29.9	0.216	50.0 ± 15.9	47.4 ± 23.2	0.104
PBV (ml/100 ml)	6.28 ± 2.8	6.12 ± 2.6	0.542	4.31 ± 1.1	4.44 ± 2.2	0.715
**LoA**						
QDP		± 14.7 ( ± 38% of the median)			±21.2 ( ± 37% of the median)	
PBF (ml/100 ml/min)		± 53.5 ( ± 89% of the median)			±26.2 ( ± 55% of the median)	
PBV (ml/100 ml)		± 3.0 ( ± 55% of the median)			±2.3 ( ± 51% of the median)	
**MID**						
QDP		5.3 (13.9% of the median)			7.7 (13.6% of the median)	
PBF (ml/100 ml/min)		19.3 (32.2% of the median)			9.5 (20.0% of the median)	
PBV (ml/100 ml)		1.1 (20.0% of the median)			0.8 (17.8% of the median)	

Minimal important differences (MID) were calculated based on standard error of measurement from repeated measures analysis of variance (rANOVA).

COPD, Chronic obstructive pulmonary disease; LoA, limits of agreement; PBF, pulmonary blood flow; PBV, pulmonary blood volume; QDP, perfusion defects in percent; SD, standard deviation.

Importantly, MID were distinctly smaller related to the median for QDP (CF: 13.9%, COPD: 13.6%) compared to PBF (CF: 32.2%, COPD: 20.0%) and PBV (CF: 20.0%, COPD: 17.8%) (Table 2).

### Quantitative perfusion parameters correlate with MRI perfusion score

**FIGURE 3:**
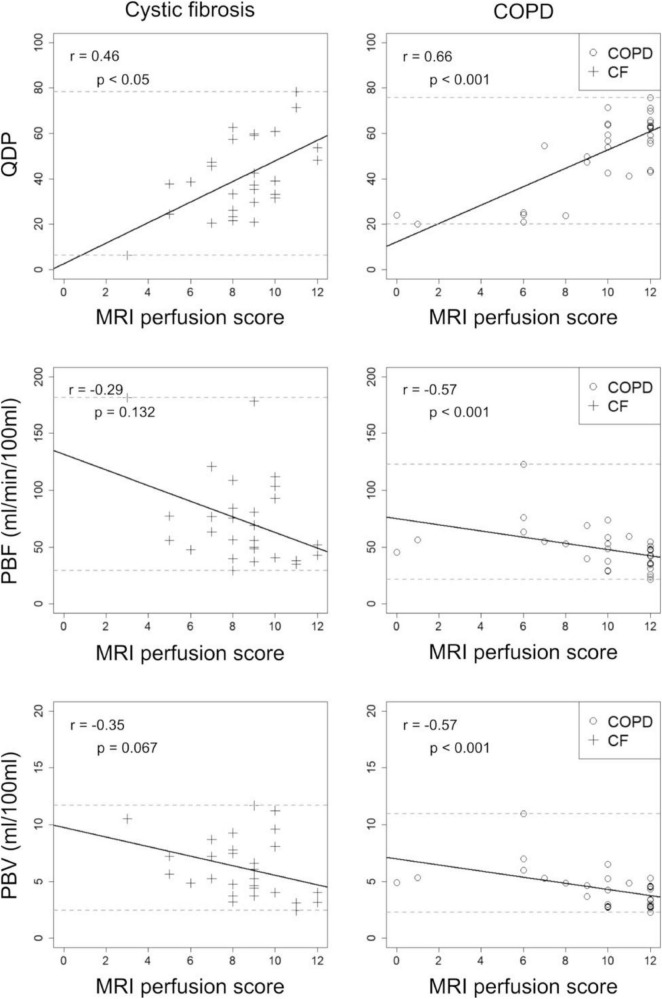
Correlations between perfusion defects in percent (QDP), pulmonary blood flow (PBF), and pulmonary blood volume (PBV) with the visual MRI perfusion score in 13 cystic fibrosis (CF, crosses) and 14 chronic obstructive pulmonary disease (COPD, circles) patients. MRI1 and MRI2 were combined. Spearman’s r correlation coefficients and corresponding *p*-values are given in each panel. Solid lines indicate the linear regression, dashed lines the minimum and maximum observed value for the parameter on the y-axis.

QDP correlated moderately with the MRI perfusion score in CF (*r* = 0.46, *p* < 0.05) and in COPD (*r* = 0.66, *p* < 0.001). In contrast, PBF and PBV correlated only poorly with the MRI perfusion score in CF (*r* = -0.29, *p* = 0.132 and *r* =−0.35, *p* = 0.067) and moderately in COPD (*r* =−0.57, *p* < 0.001 and *r* =−0.57, *p* < 0.001) (Figure 3).

In comparison, QDP correlated significantly better (Pearson and Filon’s z) with the MRI perfusion score than PBF and PBV (*p* < 0.001–0.05).

### Quantitative perfusion parameters correlate with spirometry

**FIGURE 4:**
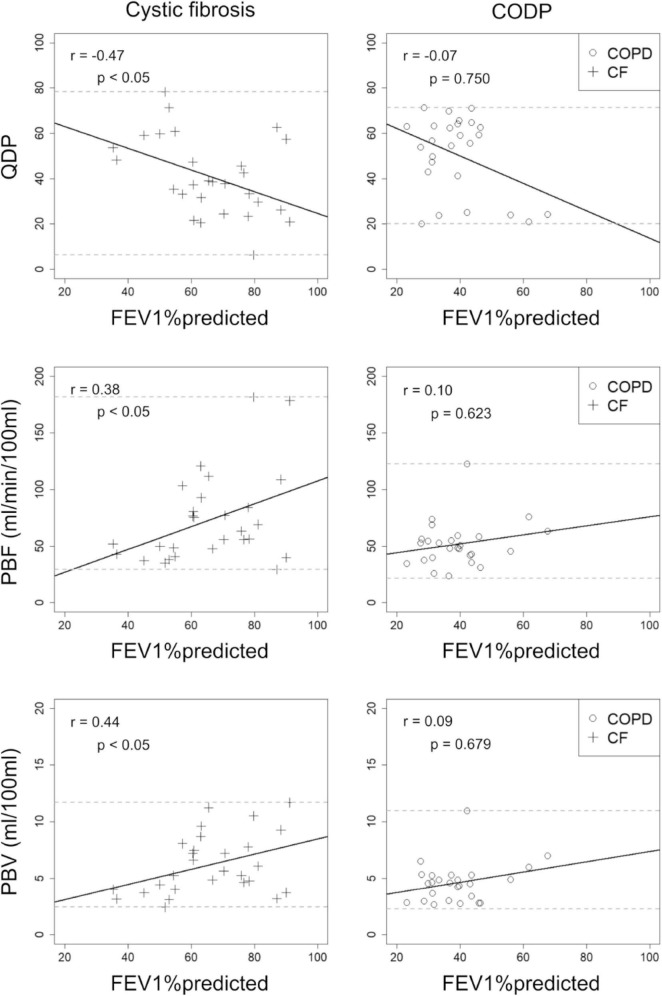
Correlations between visual MRI perfusion score, perfusion defect percent (QDP), pulmonary blood flow (PBF) and pulmonary blood volume (PBV) with forced expiratory volume in 1s percent predicted (FEV1%predicted) in 13 cystic fibrosis (CF, crosses) and post-bronchodilator FEV1%predicted in 14 chronic obstructive pulmonary disease (COPD, circles) patients. MRI1 and MRI2 were combined. Spearman’s r correlation coefficients and corresponding *p*-values are given in each panel. Solid lines indicate the linear regression lines, dashed lines the minimum and maximum observed value of the parameter on the y-axis.

Overall, higher correlations with FEV1%predicted were observed in CF compared to COPD, when combining MRI1 and MRI2. QDP and PBV correlated moderately with FEV1%predicted in CF (*r* =−0.47, *p* < 0.05 and *r* = 0.44, *p* < 0.05, respectively), but not in COPD (*r* =−0.07, *p* = 0.750 and *r* = 0.09, *p* = 0.679, respectively). PBF correlated weakly with FEV1%predicted in CF (*r* = 0.38, *p* < 0.05), but not in COPD (*r* = 0.10, *p* = 0.623) (Figure 4).

QDP did not correlate significantly better with FEV1%predicted neither compared to PBF nor to PBV (CF: *p* = 0.06 and *p* = 0.260, respectively).

## Discussion

In this midterm reproducibility study, we investigated QDP automatically quantified from DCE-MRI data using an unsupervised histogram-based clustering algorithm (Otsu’s method) in a cohort of clinically stable CF and COPD patients. Our results showed that QDP (a) is more reproducible, (b) has a stronger association with MRI perfusion score, and (c) has a stronger association with spirometry, compared to PBF and PBV.

In clinical development aiming to investigate treatments for chronic muco-obstructive lung diseases, pulmonary perfusion biomarkers could be of particular interest as perfusion changes are reversible and may precede structural, often irreversible, morphological abnormalities of the lungs (5, 34–37). In previous studies, PBF and PBV have been shown to yield a limited reproducibility. In a short-term reproducibility study (1 day between the MRI examination) with fourteen healthy subjects and manual lung segmentation, Ley-Zaporozhan et al. found significant differences between the examinations in the intra-observer comparison with LoA of about 42% of the mean PBF and with LoA of about 47% of the mean PBV (17). The short-term reproducibility study (1 day between the MRI examinations) by Ter-Karapetyan et al. in COPD patients showed LoA of approximately 46% of the observed mean PBF and 53% of the observed mean PBV (18). In our present study, we found comparable LoA related to the median in COPD for PBF and PBV. In CF, we observed higher LoA for PBF compared to COPD, which was driven by outliers. In contrast, QDP showed a higher reproducibility and a smaller MID compared to PBF and PBV in our study and the previously reported PBF and PBV reproducibility studies. In contrast to Ley-Zaporozhan et al. we investigated the reproducibility of quantitative perfusion parameters in patients with muco-obstructive lung diseases and not in healthy subjects, because pulmonary perfusion defects do not occur in healthy subjects and because different diseases may have a different influence on MRI data quality, which may affect reproducibility (17, 38).

The QDP quantification makes use of the advantages of both mathematical models based on the principles of tracer kinetics for non-diffusible tracers (39, 40) by using R(t) maps and unsupervised image clustering algorithms, i.e., in this case Otsu’s method. The use of Otsu’s method as clustering algorithm on Rmax maps was the outcome of a selection process comparing different methods, including different clustering methods, e.g., k-means clustering, 80th percentile threshold, and source images, e.g., subtraction images, R(t) maps, PBF maps, etc., as described elsewhere (12). We also tested the reproducibility of QDP computed using k-means clustering and 80th percentile threshold in this study, but the LoA were greater than for QDP computed with Otsu’s method (data not shown). Interestingly, the R(t) maps distinguish well-perfused areas from poorly perfused areas in the lungs more accurately than subtraction images. The utilization of clustering algorithms enables the assessment of all voxels relative to each other, in contrast to PBF and PBV, which evaluate the absolute voxel values separately. As a result, an intrinsic normalization of the values to each other is achieved in the QDP quantification, which leads to a quantification more robust against calculation errors affecting the whole lung equally, such as non-linearities of the contrast agent concentration to signal relation in the AIF, differences in the inspiratory level and low temporal resolution. For example, PBF and PBV calculations are more susceptible to interference caused by the low temporal resolution of DCE-MRI in the lung than QDP. The low temporal resolution leads to undersampling effects resulting in inconsistent underestimations of the AIF maximum between scans and patients. The following systematic error in the AIF maximum lead to interscan variabilities in the residue function after the deconvolution of the AIF with each lung voxel. However, since all values of the residue function within the same scan are underestimated by the same factor, the interscan variability of QDP is distinctly less affected than that of PBF and PBV. Unsupervised clustering algorithms have previously been used in Fourier decomposition-, hyperpolarized helium- and xenon-MRI to enable the assessment of ventilation or perfusion abnormalities. These studies demonstrated that the use of unsupervised clustering algorithm resulted in robust and sensitive markers of disease severity. One particular advantage of unsupervised clustering algorithms is that the algorithm does not need to be adjusted when the MR pulse sequences used are adapted/improved (9, 41–45).

Given the pathophysiologic differences between CF and COPD, a better reproducibility in CF compared to COPD is expected, which was observed for QDP but not for PBF and PBV. The underlying mucus-related disease processes in CF lead to an increase in proton density (“plus pathology”), whereas emphysema in COPD leads to a decrease in proton density (“minus pathology”) (38). We suspect that increased perfusion in CF compared to COPD causes higher calculation errors with higher outliers in the PBF and PBV quantification.

QDP detected a low but significant change between the two visits for COPD, but not PBF and PBV. The study by Wielpütz et al. which investigated the same patient population with the complete morpho-functional scoring system, also reported a significant change in the same direction for the MRI global score in COPD. It was speculated that a low but true subclinical increase in disease severity was detected by the MRI global score (7), which could also be the case for QDP in this evaluation.

In CF and COPD, QDP correlated well with the MRI perfusion score and observed correlation coefficients were higher compared to PBF and PBV. The observed correlations are in agreement with a previous cross-sectional study with 83 COPD patients from the COSYCONET cohort (12).

When correlating the quantitative MRI perfusions parameters with spirometry, we found good correlations in CF but no correlations in COPD. The observed moderate correlations between spirometry and MRI perfusion parameters in CF are in agreement with the notion that FEV1%predicted is mainly a measure of large airway obstruction, whereas perfusion abnormalities are probably mainly driven by small airways disease (5). Therefore, the observed degree of correlation between QDP and FEV1%predicted in this study for CF is as expected. Similar correlation coefficients were found in CF between FEV1%predicted and inflammatory markers in sputum assessed by MRI (46, 47). For COPD, the lack of relevant correlations in this study is most likely due to a limited range of disease severity in the evaluated COPD patients. Furthermore, emphysema, pulmonary hypertension, or peripheral obstructions have an influence on QDP in COPD, whereas FEV1%predicted is mainly driven by large airway obstructions, which may cause disagreement between QDP and FEV1%predicted (12, 48). In a previous COPD study, with a broader disease severity range, a moderate correlation between QDP and the ratio between FEV1 and forced vital capacity (FEV1/FVC) was observed (12).

## Conclusion

Major limitations of our study are the small sample size and the limited range of disease severity in the COPD population due to the recruitment of patients from our hospital’s outpatient clinic, mainly treating patients with severe COPD and emphysema. Furthermore, the reproducibility assessment in this study was limited to the use of the same scanner for baseline and follow-up examinations. A limitation regarding the QDP calculation is the strong spatial filtering of the image data, which is needed due to the present data quality, resulting in a loss of accuracy. Overall, in future clinical studies with improved DCE-MRI, the results for QDP, PBF, and PBV may improve further. QDP, PBF, and PBV calculation in the current automated pipeline can only compensate for minor breathing artifacts by removing the affected lung areas. Further development of the QDP quantification to enable the analysis of image series in free-breathing or with substantial breathing artifacts would prevent the exclusion of image data.

With this reproducibility study, we continued the technical and clinical validation of QDP, which quantifies the extent of pulmonary perfusion defects in percent from DCE-MRI using an unsupervised clustering algorithm. However, large longitudinal studies and studies evaluating the relationship between QDP and diffusing capacity of lung for carbon monoxide (DLCO) or QDP and lung clearance index (LCI) are needed before QDP can be used for clinical drug development studies in muco-obstructive lung diseases.

QDP was more robust than the previously used metrics PBF and PBV with smaller LoA and MID. As a result, smaller sample sizes and/or shorter study durations are required for QDP compared to PBF and PBV in future clinical studies to detect therapeutic response. With this study, we provide the MID of QDP derived from DCE-MRI data to support the use in future clinical drug development studies. Furthermore, since there is a relationship between ventilation and perfusion by HPV, pulmonary perfusion can be an important marker of how patients feel (44). The study results emphasize the potential of QDP as a robust, sensitive, non-invasive, and radiation-free biomarker.

## Data availability statement

The datasets presented in this article are not readily available because in adherence with the Boehringer Ingelheim Policy on Transparency and Publication of Clinical Study Data, scientific and medical researchers can request access to clinical study data after publication of the primary manuscript in a peer-reviewed journal, regulatory activities are complete and other criteria are met. Researchers should use the https://vivli.org/ link to request access to study data and visit https://www.mystudywindow.com/msw/datasharing for further information.

## Ethics statement

The studies involving human participants were reviewed and approved by the Institutional Ethics Committee (Ethik-Kommission der Medizinischen Fakultät der Universität Heidelberg, S-126/2015). The patients/participants provided their written informed consent to participate in this study.

## Author contributions

ME, HH, MP, H-UK, CH, FR, and MW contributed to study concept and design. MK, ST, ME, SB, HH, SW, RE, MP, GH, CH, FR, and MW contributed to the acquisition, analysis, and interpretation of the data. MK, ST, FR, and MW developed the new software used in this work. All authors revised and approved the final manuscript before submission.

## Abbreviations

AIF: arterial input function
CF: cystic fibrosis
CNR: contrast-to-noise ratios
COPD: chronic obstructive pulmonary disease
DCE-MRI: Dynamic contrast-enhanced magnetic resonance imaging
FEV1%predicted: forced expiratory volume in 1s percent predicted
FEV1/FVC: ratio between forced expiratory volume in 1s and forced vital capacity
LoA: limits of agreement
MID: minimal important difference
PBF: pulmonary blood flow
PBV: pulmonary blood volume
QDP: perfusion defects in percent
rANOVA: repeated measures analysis of variance
Rmax map: residue function map at the time point of maximum contrast enhancement
R(t) map: time-resolved residue function map
SD: standard deviation.
