# Supplementary material for: Unsupervised clustering algorithms improve the reproducibility of dynamic contrast-enhanced magnetic resonance imaging pulmonary perfusion quantification in muco-obstructive lung diseases
Source: Front Med (Lausanne). 2022 Oct 24;9:1022981. doi: 10.3389/fmed.2022.1022981 (PMC9637664; doi:10.3389/fmed.2022.1022981)
Supplement: Supplementary file 1 [file Data_Sheet_1.docx]

**Unsupervised clustering improves the reproducibility of DCE-MRI pulmonary perfusion quantification in muco-obstructive lung diseases**

*Online supplementary data*

**Supplemental materials and methods**

**Study inclusion and exclusion criteria**

*Cystic fibrosis inclusion criteria*

- Signed and dated informed consent consistent with ICH-GCP guidelines and local legislation prior to participation in the study
- Patients with a documented diagnosis of CF (positive sweat chloride ≥60 mg/litre, by pilocarpine iontophoresis) and/or a genotype with two identifiable mutations consistent with CF (Table S1) accompanied by one or more clinical features with the CF phenotype
- Age of ≥ 18 years
- Body mass index ≥ 18 and ≤ 30 kg/m2
- Investigator should ascertain that the patient was symptomatically stable as defined by: (i) no evidence of acute upper or lower respiratory tract infection within 2 weeks prior to first visit, and (ii) no pulmonary exacerbation requiring use of iv/oral/inhaled antibiotics, or oral corticosteroids within 2 weeks prior to first visit.
- Vital signs within the following ranges: 90-180 mmHg (SBP), 50-110 mmHg (DBP), 40- 110 bpm (pulse rate), an ear temperature within 35-37.5°C, and a GFR ≥ 30 ml/min
- Pre-bronchodilator forced expiratory volume in 1s percent predicted (FEV1%predicted) ≥ 30% at first visit, applying validated standards and reference values for lung function tests of the European Respiratory Society (ERS) and the standards of the American Thoracic Society (ATS), and the European Coal and Steal Community (ECSC) [1, 2]
- Patients who were on daily inhaled antibiotic use must be stabilized for at least 6 weeks prior to first visit
- Patients must be able to perform all study related procedures including technically acceptable pulmonary function tests

*Cystic fibrosis exclusion criteria*

- Significant pulmonary disease other than CF or other medical conditions (as determined by medical history, examination and clinical investigations at screening, e.g., cardiac, gastro-intestinal, hepatic, renal, metabolic, dermatologic, neurological, haematological, oncological, infectious, and psychiatric) that may, in the opinion of the investigator, result in any of the following: a) Put the patient at risk because of participation in the study. b) Influence the results of the study. c) Cause concern regarding the patient’s ability to participate in the study
- Previous participation in this study
- Male and non-pregnant female patients with any known hypersensitivity to Gd-based contrast agents or who do not tolerate repeated MRI scanning
- Significant alcohol or drug abuse within past two years prior to screening. See exclusion criterion No.1
- Thoracotomy and pulmonary resection prior to the study
- Planned surgery during the study
- Patients being investigated/treated by applying bronchoalveolar lavage within two days prior to or during observational period of the study (between visit 1 and visit 2)
- Women of childbearing potential not using a highly effective method of birth control. Female patients will be considered to be of childbearing potential unless surgically sterilized by hysterectomy or bilateral tubal ligation, or post-menopausal for at least two years in order to prevent pregnancy during the study period
- Planning to become pregnant during the study
- Pregnant or nursing women
- Patients who have started a new chronic medication for CF within 2 weeks before the first Visit

*COPD inclusion criteria*

- Signed and dated informed consent consistent with ICH-GCP guidelines and local legislation prior to participation in the study
- All patients must have a diagnosis of COPD according to the GOLD criteria [3] and must meet the following spirometric criteria. Patients must have stable airway obstruction with a post-bronchodilator FEV1 predicted ≥ 30% and a post-bronchodilator ratio between forced expiratory volume in 1s and forced vital capacity (FEV1/FVC) < 70% at first Visit, applying validated standards and reference values for lung function tests of the European Respiratory Society (ERS) and the standards of the American Thoracic Society (ATS), and the European Coal and Steal Community (ECSC) [1, 2]
- Age of ≥ 40 years
- Body mass index ≥ 18 and ≤ 30 kg/m^2^
- Patients must be current or ex-smokers with a smoking history of more than 10 pack years. Patients who have never smoked cigarettes must be excluded
- Vital signs within the following ranges 90-180 mmHg (SBP), 50-110 mmHg (DBP), 40 - 110 bpm (pulse rate), an ear temperature within 35-37.5°C, and a GFR ≥ 30 ml/min
- Patients must be able to perform all study related procedures including technically acceptable pulmonary function tests

*COPD exclusion criteria*

- Significant pulmonary disease other than COPD or other medical conditions (as determined by medical history, examination and clinical investigations at screening, e.g. cardiac, gastro-intestinal, hepatic, renal, metabolic, dermatologic, neurological, haematological, oncological, infectious and psychiatric) that may, in the opinion of the investigator result in any of the following: a) Put the patient at risk because of participation in the study b) Influence the results of the study c) Cause concern regarding the patient’s ability to participate in the study
- Previous participation in this study
- Patients with malignancy for which the patient has undergone resection, radiation or chemotherapy within past one year. Patients with treated basal cell carcinoma or fully cured squamous cell carcinoma were allowed
- Male and non-pregnant female patients with any known hypersensitivity to Gd-based contrast agents or who do not tolerate repeated MRI scanning
- A history of life-threatening pulmonary obstruction
- Thoracotomy and pulmonary resection prior to the study
- Planned surgery during the study
- Patients being investigated/treated by applying bronchoalveolar lavage within two days prior o or during observational period of the study (between Visit 1 and Visit 2)
- Respiratory tract infection or COPD exacerbation in the 2 weeks prior to first Visit.
- Known active tuberculosis
- Significant alcohol or drug abuse within past 2 years prior to screening. See exclusion criterion No.1
- Women of childbearing potential not using a highly effective method of birth control. Female patients will be considered to be of childbearing potential unless surgically sterilized by hysterectomy or bilateral tubal ligation, or post-menopausal for at least two years
- Pregnant or nursing women
- Patients being treated with oral corticosteroid medication at unstable doses (i.e., less than 6 weeks on a stable dose prior to screening)

**Medication, worsening of the clinical condition, exacerbation and patient exclusion**

For pulmonary exacerbations in CF, the previously described criteria were applied [4, 5]. For COPD, criteria published by the GOLD consortium were applied [3]. Changes in vital signs, ECG, physical examination and laboratory test results were also considered by the investigator. In general, subjects were allowed to and also should continue their usual care and medication. There was no medication restricted from being used over the complete trial period.

An individual subject, CF and COPD, is to be withdrawn from the study if any of the following criteria apply:

- The subject withdraws consent.
- Administrative reasons (Protocol violations, persistent non-compliance)
- The subject is no longer able to participate for medical reasons, e.g. severe exacerbation of the disease between MRI1 and MRI2, surgery, adverse events (especially to Gadolinium-based contrast after MRI1), other diseases, or positive pregnancy test.

During the study, patients should maintain their baseline therapy regimes. Allowed additional treatments included:

- Salbutamol (albuterol) inhalation aerosol to control for temporary changes in symptoms
- Temporary increases in the dose or addition of oral steroids were allowed. MRI1 and 2 should not occur within seven days of the last administered dose of an increase or addition of oral steroids. MRI1 or 2 may be postponed up to seven days to accommodate this restriction
- Temporary additions of theophylline preparations were allowed. MRI1 and 2 should not occur within seven days of the last dose. MRI1 or 2 may be postponed up to seven days to accommodate this restriction
- The use of antibiotics in addition to baseline antibiotic treatments (e.g. inhalative tobramycin) is not restricted and may be prescribed as medically necessary for exacerbations between MRI1 and MRI2. MRI1 and 2 should not occur within seven days of the last dose of additional antibiotics for exacerbation. MRI1 and MRI2 may be postponed up to two days to accommodate this restriction

Violations against abovementioned medication regime led to study removal.

**Pulmonary function testing and clinical assessment**

*Spirometry*

Spirometry was conducted with the subject in a seated position (MasterScreen Body, E. Jaeger, Hoechberg, Germany). The best of three efforts (with a maximum of eight attempts) defined as the highest forced expiratory volume in 1s (FEV1) and the highest forced vital capacity (FVC) each obtained on any of the three blows were used.

There were no restrictions regarding concomitant treatment and lifestyle except restriction prior to pulmonary function testing that were required to standardize lung function values obtained during the trial:

- At least 1 hour for the morning dose of inhaled steroids
- At least 8-hours for short-acting beta-adrenergic bronchodilators
- At least 12 hours for long-acting beta-adrenergic bronchodilators
- At least 24 hours for long-acting beta-adrenergic bronchodilators
- At least 8 hours for short-acting anticholinergic bronchodilators
- At least 24 hours for long-acting anticholinergic bronchodilators
- At least 12 hours for short-acting theophylline preparations
- At least 24 hours for long-acting theophylline preparation
- At least 24 hours for leukotriene modifiers (e.g., montelukast)
- The subject must remain in the building where the pulmonary function testing was performed and must return to the laboratory at least ten minutes prior to the start of each test
- On pulmonary function test days (MRI1 and MRI2), subjects must refrain from strenuous activity for at least twelve hours prior to pulmonary function testing and throughout the testing period. Subjects should also avoid cold temperatures, environmental smoke, dust or areas with strong odours (e.g., perfumes)
- Coffee, tea, chocolate, cola and other caffeine-containing beverages and foods, and ice-cold beverages were not allowed at least three hours prior to pulmonary function testing
- Smoking should be discouraged for twelve hours prior to pulmonary function testing and will not be permitted on the test day until completion of all study-related examinations on MRI1 and MRI2, respectively. However, if the patient still had a smoke his / her spirometry data should anyway be retained for further analysis

*Clinical examination*

Pulse rate, blood pressure and body temperature were measured and recorded at screening and prior to pulmonary function testing on MRI1 and MRI2, with the subject seated and rested for a minimum of five minutes. A physical examination was conducted at screening to determine in- and exclusion criteria. The purpose of the physical examination at MRI1 was to obtain information about the subject’s baseline condition that may have not been elicited in obtaining the medical history. At MRI2, the physical examination was conducted to capture the subject´s condition at the end of the observational period of the study. Significant changes to the physical examination were evaluated and reported (see removal criteria as stated above).

Microbiological evaluation in CF patients in our outpatient clinic was performed on throat swabs and induced sputum routinely every three months. Pseudomonas aeruginosa infection status was defined according to the following criteria as negative, intermittent or chronic infection as previously described [6–8]: Patients were categorized as *P. aeruginosa* negative, when there was no growth of *P. aeruginosa* in the previous twelve months and titres of precipitating antibodies against *P. aeruginosa* antigens (alkaline protease, elastase and exotoxin A; Mediagnost, Reutlingen, Germany) were negative. Intermittent *P. aeruginosa* infection was defined as culturing of *P. aeruginosa* in less than 50% of the samples in the last twelve months and negative antibodies. Chronic *P. aeruginosa* infection was defined as persistent presence of *P. aeruginosa* for at least 6 consecutive months or less when combined with increased levels (titre >1:1250) of two or more *P. aeruginosa* antibodies. Infection status is summarized in Table 1 in the main manuscript.

The smoking status (current smoker or ex-smoker) and number of pack years of each subject were recorded at screening. All active and relevant historical disorders were recorded at MRI1. Prior to pulmonary function testing, the time of the last cigarette smoked was recorded if the subject had smoked a cigarette during the twelve hours prior to the start of the lung function measurement procedures.

All concomitant therapies taken in the six-weeks period preceding screening and throughout the observation period including the follow-up visit were recorded.

All adverse events were recorded incl. those related to gadolinium administered to the patient as per protocol during MRI1 and 2, results of clinically relevant abnormalities found at physical examination, vital signs or ECG that were not pre-existing prior to signing of informed consent (study inclusion). Patients were followed by a standardized telephone interview one week (7 ± 1 days) after MRI2, again assessing adverse events and changes in clinical condition.

*Laboratory testing*

Haematology, blood chemistry, and urinalysis were conducted each at screening, MRI1 and MRI2. Haematology: Haemoglobin, haematocrit, red blood cell count, white blood cell count including differential (neutrophils, lymphocytes, monocytes, eosinophils, basophils), total eosinophil count and platelet count. Blood chemistry: Alkaline phosphatase, LDH, Gamma-GT, SGOT, SGPT, glucose, calcium, inorganic phosphate, uric acid, urea nitrogen, creatinine, total protein, potassium, sodium, chloride, total bilirubin, creatine phosphokinase (CPK). Coagulation parameters: International normalized ratio (INR) / prothrombin time (PT), partial thromboplastin time (PTT). Urinalysis: Specific gravity, pH, glucose, protein, occult blood. Urine pregnancy testing was executed on all females of child-bearing potential, at MRI1 and MRI2 prior to any blood sampling or MRI examination.

*Electrocardiogram*

A standard twelve-lead electrocardiogram (ECG) was performed on all subjects at MRI1 and 2.

**Magnetic resonance imaging**

A T1-weighted gradient echo sequence (GRE) and first-pass dynamic contrast-enhanced magnetic resonance imaging (DCE-MRI) were acquired using a clinical 1.5T MRI scanner (Magnetom Aera, Siemens Healthineers, Erlangen, Germany) as previously described [9–12]. The protocol is summarized in Table S2. The DCE-MRI acquisition started before and continued during intravenous contrast injection with 20 acquisitions and a temporal resolution of 1.63 s. Gadolinium-based CA (Gadobutrol, Bayer Vital GmbH, Leverkusen, Germany) with a dose less than 0.1 mmol/kg body weight was injected at a rate of 3 – 5 ml/s with a power injector. Contrast agent dose was chosen low enough to obtain a pseudolinear dependence of the of the contrast agent concentration to signal relationship in the lung parenchyma, but not in the input function of the quantification (AIF) [13–15]. All sequences were acquired in inspiratory breath-hold. Of note, MRI scanning was performed after patients had taken their maintenance medication, as opposed to spirometric lung function testing.

**Statistical analysis**

By convention, r between 0.0-0.2 was regarded as negligible, 0.2-0.4 as weak, 0.4-0.7 moderate, 0.7-0.9 strong, and 0.9-1.0 very strong correlation[16]. The Kruskal-Wallis test was used for comparison between CF and COPD groups. Bland-Altman plots and Wilcoxon signed-rank test were used for intra-group comparisons of MRI2 with MRI1. Correlation coefficients were compared statistically with the R-package “cocur” [17] and the p-value from Pearson and Filon's z from the package was used [18].

**Table S1** Summary of *CFTR* genotypes with pancreatic status of subjects with cystic fibrosis

| **Pancreatic insufficient** | | **Pancreatic sufficient** | |
| --- | --- | --- | --- |
| ***CFTR* genotype** | **Number of**  **subjects n** | ***CFTR* genotype** | **Number of**  **subjects n** |
|  |  |  |  |
| F508del / F508del | 8 | F508del / p. T1299I | 1 |
| F508del / I507del | 2 | G542X / X | 1 |
| F508del / R347P | 1 |  |  |
| F508del / CFTRdele17 (2.5 kb) | 1 |  |  |
| 1717-1G>A / Q552X | 1 |  |  |

**Table S2**  Standardized MRI protocol [19]

| **Sequence** | **Specials** | **Plane** | **TR (ms)** | **TE (ms)** | **ST (mm)** | **Distance factor (%)** | **Slices** | **FoV (mm^2^)** | **Matrix** | **Voxel size (mm)** | **Scan time (min:s)** |
| --- | --- | --- | --- | --- | --- | --- | --- | --- | --- | --- | --- |
| T1 GRE | Bh | tra | 3.61 | 1.69 | 4.0 | 3D | 88 | 400x300 | 320x240 | 1.3 x 1.3 | 0:18 |
| T1 GRE | Bh | cor | 3.35 | 1.63 | 4.0 | 3D | 56 | 400x400 | 288x288 | 1.4 x 1.4 | 0:16 |
| DCE-MRI | 20 acquisitions, 1 bh, Gd | cor | 1.80 | 0.74 | 5.0 | 3D | 44 | 450x366 | 256x208 | 1.8 x 1.8 | 0:38 |
| T1 GRE = T1-weighted gradient echo sequence (GRE), volume interpolated breath-hold acquisition (VIBE), 4D Perfusion = time-resolved 3D gradient echo sequence with parallel imaging and view sharing, time-resolved angiography with stochastic trajectories (TWIST), Gd = i.v. injection of Gadolinium-based contrast material, tra = transversal plane, cor = coronary plane, bh = breath-hold, TR = repetition time, TE = echo time, ST = slice thickness, FoV = Field of view. | | | | | | | | | | | |

**Supplemental references**

1. Quanjer PH, Tammeling GJ, Cotes JE, Pedersen OF, Peslin R, Yernault JC (1993) Lung volumes and forced ventilatory flows. Report Working Party Standardization of Lung Function Tests, European Community for Steel and Coal. Official Statement of the European Respiratory Society. Eur Respir J Suppl 16:5–40

2. Miller MR (2005) Standardisation of spirometry. Eur Respir J 26:319–338. https://doi.org/10.1183/09031936.05.00034805

3. Singh D, Agusti A, Anzueto A, Barnes PJ, Bourbeau J, Celli BR, Criner GJ, Frith P, Halpin DMG, Han M, Varela MVL, Martinez F, Oca MM de, Papi A, Pavord ID, Roche N, Sin DD, Stockley R, Vestbo J, Wedzicha JA, Vogelmeier C (2019) Global Strategy for the Diagnosis, Management, and Prevention of Chronic Obstructive Lung Disease: the GOLD science committee report 2019. Eur Respir J 53:1900164. https://doi.org/10.1183/13993003.00164-2019

4. Fuchs HJ, Borowitz DS, Christiansen DH, Morris EM, Nash ML, Ramsey BW, Rosenstein BJ, Smith AL, Wohl ME (1994) Effect of Aerosolized Recombinant Human DNase on Exacerbations of Respiratory Symptoms and on Pulmonary Function in Patients with Cystic Fibrosis. New Engl J Medicine 331:637–642. https://doi.org/10.1056/nejm199409083311003

5. Rosenfeld M, Ratjen F, Brumback L, Daniel S, Rowbotham R, McNamara S, Johnson R, Kronmal R, Davis SD, Group for the IS (2012) Inhaled Hypertonic Saline in Infants and Children Younger Than 6 Years With Cystic Fibrosis: The ISIS Randomized Controlled Trial. Jama 307:2269–2277. https://doi.org/10.1001/jama.2012.5214

6. Hoiby N, Flensborg EW, Beck B, Friis B, Jacobsen SV, Jacobsen L (1977) Pseudomonas aeruginosa infection in cystic fibrosis. Diagnostic and prognostic significance of Pseudomonas aeruginosa precipitins determined by means of crossed immunoelectrophoresis. Scand J Respir Dis 58:65–79

7. Pressler T, Bohmova C, Conway S, Dumcius S, Hjelte L, Høiby N, Kollberg H, Tümmler B, Vavrova V (2011) Chronic Pseudomonas aeruginosa infection definition: EuroCareCF Working Group report. J Cyst Fibros 10:S75–S78. https://doi.org/10.1016/s1569-1993(11)60011-8

8. Boutin S, Graeber SY, Weitnauer M, Panitz J, Stahl M, Clausznitzer D, Kaderali L, Einarsson G, Tunney MM, Elborn JS, Mall MA, Dalpke AH (2015) Comparison of Microbiomes from Different Niches of Upper and Lower Airways in Children and Adolescents with Cystic Fibrosis. Plos One 10:e0116029. https://doi.org/10.1371/journal.pone.0116029

9. Eichinger M, Optazaite DE, Kopp-Schneider A, Hintze C, Biederer J, Niemann A, Mall MA, Wielputz MO, Kauczor HU, Puderbach M (2012) Morphologic and functional scoring of cystic fibrosis lung disease using MRI. Eur J Radiol 81:1321–9. https://doi.org/10.1016/j.ejrad.2011.02.045

10. Jobst BJ, Wielpütz MO, Triphan SMF, Anjorin A, Ley-Zaporozhan J, Kauczor H-U, Biederer J, Ley S, Sedlaczek O (2015) Morpho-Functional 1H-MRI of the Lung in COPD: Short-Term Test-Retest Reliability. Plos One 10:e0137282. https://doi.org/10.1371/journal.pone.0137282

11. Wielpütz M, Eichinger M, Biederer J, Wege S, Stahl M, Sommerburg O, Mall M, Kauczor H, Puderbach M (2016) Imaging of Cystic Fibrosis Lung Disease and Clinical Interpretation. R Fo - Fortschritte Auf Dem Gebiet Der R Ntgenstrahlen Und Der Bildgebenden Verfahren 188:834–845. https://doi.org/10.1055/s-0042-104936

12. Stahl M, Wielpütz MO, Graeber SY, Joachim C, Sommerburg O, Kauczor H-U, Puderbach M, Eichinger M, Mall MA (2016) Comparison of Lung Clearance Index and Magnetic Resonance Imaging for Assessment of Lung Disease in Children with Cystic Fibrosis. Am J Resp Crit Care 195:349–359. https://doi.org/10.1164/rccm.201604-0893oc

13. Neeb D, Kunz RP, Ley S, Szábo G, Strauss LG, Kauczor H, Kreitner K, Schreiber LM (2009) Quantification of pulmonary blood flow (PBF): Validation of perfusion MRI and nonlinear contrast agent (CA) dose correction with H 215O positron emission tomography (PET). Magnet Reson Med 62:476–487. https://doi.org/10.1002/mrm.22025

14. Pintaske J, Martirosian P, Graf H, Erb G, Lodemann K-P, Claussen CD, Schick F (2006) Relaxivity of Gadopentetate Dimeglumine (Magnevist), Gadobutrol (Gadovist), and Gadobenate Dimeglumine (MultiHance) in Human Blood Plasma at 0.2, 1.5, and 3 Tesla. Invest Radiol 41:213–221. https://doi.org/10.1097/01.rli.0000197668.44926.f7

15. Puderbach M, Risse F, Biederer J, Ley-Zaporozhan J, Ley S, Szabo G, Semmler W, Kauczor H-U (2008) In vivo Gd-DTPA concentration for MR lung perfusion measurements: Assessment with computed tomography in a porcine model. Eur Radiol 18:2102–2107. https://doi.org/10.1007/s00330-008-0974-1

16. Karlik SJ (2003) Exploring and Summarizing Radiologic Data. Am J Roentgenol 180:47–54. https://doi.org/10.2214/ajr.180.1.1800047

17. Diedenhofen B, Musch J (2015) cocor: A Comprehensive Solution for the Statistical Comparison of Correlations. Plos One 10:e0121945. https://doi.org/10.1371/journal.pone.0121945

18. Pearson K, Filon LNG (1898) Mathematical contributions to the theory of evolution. IV. On the probable errors of frequency constants and on the influence of random selection on variation and correlation. P R Soc London 62:173–176. https://doi.org/10.1098/rspl.1897.0091

19. Triphan SMF, Biederer J, Burmester K, Fellhauer I, Vogelmeier CF, Jörres RA, Kauczor H-U, Heußel CP, Wielpütz MO, Jobst BJ (2018) Design and application of an MR reference phantom for multicentre lung imaging trials. Plos One 13:e0199148. https://doi.org/10.1371/journal.pone.0199148
